# Discordance Between Self-Reported Physical Activity and Field-Assessed Functional Capacity Among University Students: An Agreement and Directional Discordance Analysis

**DOI:** 10.3390/healthcare14142159

**Published:** 2026-07-17

**Authors:** Ahmad Salman

**Affiliations:** Department of Public Health Practice, College of Public Health, Kuwait University, Kuwait City 13110, Kuwait; ahmad.salman@ku.edu.kw

**Keywords:** physical activity assessment, cardiorespiratory fitness, health markers, IPAQ, Incremental Shuttle Walk Test, directional discordance, agreement analysis, weighted kappa, non-communicable diseases, Kuwait

## Abstract

**Highlights:**

**What are the main findings?**

- Self-reported physical activity (IPAQ-SF) showed only slight classification agreement with sample-derived ISWT functional-capacity tertiles (unweighted κ = 0.083; linear-weighted κ = 0.137; quadratic-weighted κ = 0.192), with matching IPAQ-ISWT category assignment in 38.8% (151/389) of university students.
- Directional discordance was sex-patterned: lower-than-tertile IPAQ classification predominated overall (42.9%), while males were more likely to show higher-than-tertile classification (31.5% vs. 14.1%) and females lower-than-tertile classification (44.1% vs. 39.1%).

**What are the implications of the main findings?**

- A statistically significant group-level association does not imply agreement between IPAQ-SF categories and field-based functional-capacity tertiles at the individual level.
- Accessible field-based functional-capacity assessment such as the ISWT may complement self-report tools by adding performance-based information to university health assessment and surveillance research.

**Abstract:**

**Background/Objectives**: Self-reported physical activity is widely used in non-communicable disease (NCD) surveillance, yet its individual-level agreement with field-based markers of cardiorespiratory fitness remains poorly characterised in Gulf Cooperation Council (GCC) populations. This study examined the association, agreement, and directional discordance between self-reported physical activity (IPAQ Short Form, IPAQ-SF) and field-assessed functional capacity (Incremental Shuttle Walk Test, ISWT) among university students in Kuwait. **Methods**: In 389 students (76.3% female; mean age 20.2 ± 2.3 years) in Kuwait, IPAQ-SF activity was classified as low, moderate, or high and ISWT distance into sample-derived tertiles. Spearman correlations, Kruskal–Wallis tests with Dunn pairwise comparisons, cross-tabulation, and unweighted, linear-weighted, and quadratic-weighted Cohen’s kappa were used. **Results**: IPAQ activity showed a weak but significant correlation with ISWT distance (ρ = 0.209, *p* < 0.001). ISWT performance did not differ between the low- and moderate-IPAQ categories (Bonferroni-adjusted Dunn p = 0.162). Agreement was slight across all kappa estimates (unweighted κ = 0.083; linear-weighted κ = 0.137; quadratic-weighted κ = 0.192), with matching category assignment in only 38.8% of participants; lower-than-tertile IPAQ classification occurred in 42.9% and higher-than-tertile in 18.3%. Directional discordance differed significantly by sex (*p* < 0.001). A rank-based partial correlation adjusting for BMI, age, and sex was attenuated but remained significant (*r* = 0.144, *p* = 0.004). **Conclusions**: IPAQ-SF categories showed poor individual-level agreement with sample-derived ISWT functional-capacity tertiles among university students in Kuwait. Self-reported physical activity and field-based functional capacity should therefore be interpreted as complementary rather than interchangeable measures. Larger multi-university studies across the GCC are needed to confirm these findings.

## 1. Introduction

Physical activity is a cornerstone of health promotion and non-communicable disease (NCD) prevention, with strong evidence linking regular participation to reduced risk of cardiovascular disease, type 2 diabetes, obesity, and premature mortality [1,2]. Among young adults, particularly university students, maintaining adequate physical activity levels is important for both immediate and long-term health, as well as for establishing behavioural patterns that influence cardiometabolic trajectories across the lifespan [3,4]. Accurate assessment of physical activity and fitness in this population is therefore essential for surveillance, risk stratification, and the design of targeted interventions.

Self-reported questionnaires remain the most widely used tools for physical activity assessment in epidemiological research due to their low cost, feasibility, and scalability [5]. The International Physical Activity Questionnaire (IPAQ) short form is among the most extensively applied instruments globally, offering standardised classification of individuals into low-, moderate-, and high-physical-activity categories [6]. However, self-reported physical activity is inherently subject to recall bias, social desirability effects, and misinterpretation of activity intensity, which may introduce both random and systematic measurement error [7,8]. Importantly, self-reported physical activity and cardiorespiratory fitness (CRF) represent related but conceptually distinct constructs: the IPAQ captures self-perceived participation in structured and incidental activity, whereas CRF reflects the integrated physiological adaptations to habitual activity, body composition, and other biological factors [9,10]. These constructs are associated but not interchangeable, and this distinction has direct implications for their use in individual-level risk assessment.

Accumulating evidence indicates that the validity of the IPAQ-SF against objective criteria is limited. Systematic reviews and meta-analyses report that correlations between IPAQ-derived total physical activity and objective measures typically fall in a low-to-modest range, and that the instrument may overestimate or misclassify physical activity relative to objective measures [11,12]. Recent evidence in university students reinforces this concern: a 2026 study of 552 students in Germany using laboratory cardiopulmonary exercise testing reported that IPAQ contributed only a small share of variance in measured VO_2_peak (R^2^ ≈ 0.02) and was a substantially weaker surrogate marker for cardiorespiratory fitness than other simple field measures [13]. Critically, a statistically significant association does not imply agreement at the individual level, nor does it ensure accurate classification into meaningful activity or fitness categories—a distinction with important consequences when self-report instruments are used to assign individuals to activity or risk categories for clinical or public health purposes [14]. Studies in adult populations have further shown that misreporting appears to be systematic rather than random, with the direction and magnitude of discordance varying across populations and fitness levels; the pattern most commonly described in non-GCC adult populations is overestimation by less fit individuals, with fitter individuals tending to underreport relative to objective measures [15,16,17,18]. Whether the same pattern holds in young adult GCC populations, where structural and cultural determinants of activity differ markedly from Western settings [19], has not been formally examined. Despite this, few studies have formally evaluated classification agreement and directional discordance between IPAQ categories and ISWT-derived fitness rankings, and virtually none have done so in Gulf Cooperation Council (GCC) populations.

This gap is particularly consequential in the GCC context. Kuwait and neighbouring countries face disproportionately high NCD burdens—with physical inactivity prevalence among the highest globally and NCDs accounting for the majority of deaths—yet physical activity surveillance relies predominantly on self-report questionnaires whose classification accuracy in these populations remains insufficiently examined [20,21]. Recent regional evidence further indicates that women in GCC settings face distinct cultural and structural barriers to physical activity participation, including limited access to facilities and other contextual constraints [19], which may affect how activity is undertaken. University students represent a strategically important group for early intervention, as this life stage is associated with marked declines in structured physical activity and the consolidation of behavioural patterns that track into adulthood [3,22]. Understanding whether self-reported physical activity categories accurately reflect field-assessed functional capacity status in this population has direct implications for how surveillance data should be interpreted and acted upon.

The Incremental Shuttle Walk Test (ISWT) is a standardised, externally paced, progressive field test that provides a valid and reproducible estimate of functional exercise capacity without requiring laboratory equipment, making it well suited to population-based fitness assessment [23,24]. The ISWT was selected for this study because it is feasible to administer in non-clinical university settings, requires no specialist equipment, and has documented criterion validity against laboratory-measured VO_2_max in adult populations [25]; we use it here as a field-based functional capacity marker rather than a direct gold-standard measure of CRF. Both IPAQ-SF and ISWT are widely used as practical markers of health in adult populations—the former capturing self-perceived activity participation, the latter reflecting performance-based field-assessed functional capacity—yet the extent to which these two health markers agree at the individual classification level has rarely been formally evaluated, particularly in young GCC populations. We hypothesised that (i) IPAQ-derived categories would show only a weak positive association with ISWT distance; (ii) individual-level classification agreement between IPAQ categories and ISWT tertiles would be poor; and (iii) directional discordance would be present and differ by sex. Using the ISWT as the field-based comparison measure, the present study aimed to examine: (i) the strength of association between IPAQ-derived physical activity categories and field-assessed functional capacity; (ii) the degree of classification agreement between IPAQ categories and ISWT-based fitness tertiles at the individual level; and (iii) the prevalence, direction, and sex-specific patterns of directional discordance.

## 2. Materials and Methods

### 2.1. Study Design and Setting

This was a cross-sectional study conducted at a public university in Kuwait. Data collection took place on campus using a standardised in-person data collection protocol from 28 July 2025 to 4 December 2025, spanning the pre-semester and fall-semester data collection period of the 2025–2026 academic year. The present study is part of a broader cross-sectional investigation of physical activity and fitness among university students. While related analyses have examined determinants of fitness outcomes, this study specifically focuses on agreement and directional discordance between self-reported physical activity and field-assessed functional capacity. The study was conducted in accordance with the Declaration of Helsinki, and ethical approval was obtained from the Health Sciences Centre Ethics Committee, Kuwait University (Approval No. VDR/EC-2025-114; approved 26 May 2025). Written informed consent was obtained from all participants prior to enrolment.

### 2.2. Participants

Participants were undergraduate and postgraduate students enrolled at the university. Recruitment was undertaken using a convenience sampling approach across faculties on the main campus; students were approached during campus-based recruitment sessions and through faculty-distributed announcements. The achieved sex distribution (76.3% female) arose from this convenience recruitment; recruitment was not sex-stratified, and invitation and refusal counts by sex were not recorded, so the study cannot determine whether the imbalance reflected the composition of the accessible student population, differential participation, or both. The sample was predominantly Kuwaiti (80.2%); the dataset records only Kuwaiti versus non-Kuwaiti status and not specific countries of origin. Eligibility was determined using the Physical Activity Readiness Questionnaire (PAR-Q+), which screened for conditions requiring exclusion from, or further evaluation before, exercise testing. Students were eligible if they were aged 18 years or older, able to understand the study procedures, and free from any condition precluding safe exercise testing. Exclusion criteria included any affirmative response to the PAR-Q+, acute musculoskeletal injury, pregnancy, or physician-advised restriction from physical activity. There were no exclusions based on sex, nationality, body weight, or academic year. All participants included in the final analytic dataset had PAR-Q+ total scores of zero. The final analytic dataset comprised 389 participants.

### 2.3. Self-Reported Physical Activity: IPAQ

Habitual physical activity was assessed using the International Physical Activity Questionnaire—Short Form (IPAQ-SF) [6], a self-administered instrument that captures the frequency, duration, and intensity of vigorous-intensity, moderate-intensity, and walking activity performed during the preceding seven days, as well as weekday sitting time. Total physical activity was expressed in metabolic equivalent task minutes per week (MET-min/week) using the standard IPAQ scoring protocol [26]. IPAQ-SF data were processed using the published IPAQ data-cleaning rules and standard category thresholds [26]. Specifically: (i) reported durations below 10 min within an activity domain were recoded to zero; (ii) duration for each intensity domain was truncated at 180 min per day (“truncation at three hours”) to limit the influence of implausibly high values; and (iii) participants reporting more than 16 h per day across all activities would be excluded as implausible. In the present sample, three participants had at least one reported activity duration below 10 min within a domain and two had at least one reported duration above 180 min/day (these durations were recoded/truncated accordingly), and no participant exceeded the 16 h/day total; all 389 records therefore remained eligible and constituted the final analytic sample. IPAQ-SF categories (low, moderate, high) were assigned using the standard IPAQ classification thresholds incorporating activity type, reported days, duration, and total MET-min/week. Because the short form records walking, moderate-intensity, and vigorous-intensity activity days separately and does not identify overlap between domains on the same calendar day, the combined-day criteria of the classification algorithm were operationalised as the capped sum of reported domain-specific activity days, limited to seven days. Participants were classified into low, moderate, and high categories according to standard IPAQ scoring criteria based on total MET-min/week and activity patterns [26]. This three-category ordinal variable was used in all agreement and group comparison analyses.

### 2.4. Field-Assessed Functional Capacity: Incremental Shuttle Walk Test

Functional exercise capacity was assessed using the Incremental Shuttle Walk Test (ISWT), a performance-based field marker related to cardiorespiratory fitness rather than a direct measurement of VO_2_max. The ISWT was originally developed by Singh et al. [23]. The test was conducted along a 10-metre flat course marked by two cones, using a standardised audio signal to dictate walking pace. Speed increased progressively each minute according to the standard protocol until participants were unable to maintain the required pace or voluntarily stopped. The total number of completed shuttles was recorded, and ISWT distance (metres) was calculated as the number of shuttles multiplied by 10. All test administrators were trained and standardised in protocol delivery prior to data collection. We selected the ISWT because it is feasible to administer in university settings, requires no laboratory equipment, and a systematic review of field-based fitness tests has confirmed its criterion validity against laboratory-measured VO_2_max across diverse adult populations [25]. Throughout this paper, ISWT-based classification is therefore framed as a field-assessed fitness marker rather than a definitive objective standard of physical fitness. For agreement analyses, ISWT distance was classified into sample-derived fitness tertiles using rank-based tertile classification across the full sample (i.e., not separately by sex), producing approximately equal-sized groups used as the comparison classification for IPAQ categories. We acknowledge that sample-derived tertiles are by construction sample-dependent and do not correspond to externally validated clinical fitness categories; the rationale for using them is to provide three internally balanced strata for comparison with IPAQ’s three-category structure.

### 2.5. Statistical Analysis

All statistical analyses were conducted using IBM SPSS Statistics version 31.0.1.0 (IBM Corp., Armonk, NY, USA). Continuous variables are presented as means ± standard deviations (SDs), with medians and interquartile ranges (IQRs) reported for non-normally distributed variables. Categorical variables are presented as frequencies and percentages. Normality was assessed using the Shapiro–Wilk test and visual inspection of histograms and normal probability plots.

Spearman rank correlations were used to examine bivariate associations between IPAQ-derived MET-min/week (total and by intensity domain) and ISWT distance, given the non-normal distributions of both variables. Asymptotic 95% confidence intervals for Spearman’s rho were computed via Fisher’s z-transformation. Sex-stratified Spearman correlations were additionally computed as exploratory subgroup analyses to examine whether the IPAQ–ISWT relationship differed by sex. Differences in ISWT distance across IPAQ categories were examined using the Kruskal–Wallis test, given violated normality and homogeneity of variance assumptions, with Bonferroni-adjusted Dunn pairwise comparisons as the post-hoc test.

Classification agreement between IPAQ categories and ISWT tertiles was examined using cross-tabulation and Cohen’s kappa. Because both IPAQ categories and ISWT tertiles are ordinal, we report Cohen’s unweighted kappa as a strict exact-agreement estimate, Cohen’s linear-weighted kappa as the primary ordinal agreement estimate, and Cohen’s quadratic-weighted kappa as an additional sensitivity analysis. Linear weights assign partial credit to adjacent-category agreement while retaining a proportional penalty for more distant disagreement; quadratic weights penalise larger category disagreements (e.g., Low vs. High) more steeply [27]. All kappa coefficients were computed from the IPAQ-SF × ISWT tertile cross-tabulation using a custom script executed in the Python 3.10 environment integrated within IBM SPSS Statistics version 31.0.1.0; 95% confidence intervals were estimated using percentile bootstrap resampling (10,000 resamples; fixed random seed for reproducibility). Kappa values were interpreted using standard benchmarks [28], with values close to zero indicating minimal agreement beyond chance.

Directional discordance was defined operationally as the difference between each participant’s IPAQ category and their ISWT tertile rank. Participants were classified as having a matching category assignment (difference = 0), a higher-than-tertile IPAQ classification (IPAQ category > ISWT tertile), or a lower-than-tertile IPAQ classification (IPAQ category < ISWT tertile). The proportion of participants in each category was computed, and sex differences in directional discordance were examined using chi-square tests. Statistical significance was set at *p* < 0.05 (two-tailed).

Exploratory sensitivity analyses examined whether the association between IPAQ total MET-min/week and ISWT distance persisted after adjustment for age, sex, and body mass index (BMI). A rank-based partial correlation was estimated, and Spearman correlations were additionally calculated within predefined BMI categories (<25, 25.0–29.9, and ≥30 kg/m^2^). BMI was calculated from measured height and weight and was available for all 389 participants. The BMI-stratified correlations were interpreted descriptively because no formal interaction tests were performed.

## 3. Results

### 3.1. Sample and Physical Activity Profile

A total of 389 students were included in the final analytic sample (297 female, 76.3%; 92 male, 23.7%), with a mean age of 20.2 ± 2.3 years. The majority were Kuwaiti nationals (312, 80.2%). The distribution of nationality did not differ by sex: among Kuwaiti participants, 237 (76.0%) were female and 75 (24.0%) male, and among non-Kuwaiti participants, 60 (77.9%) were female and 17 (22.1%) male (χ^2^(1) = 0.131, *p* = 0.717). All participants met data quality criteria.

Total physical activity was highly right-skewed (Shapiro–Wilk *p* < 0.001), with a median of 540 MET-min/week (IQR 198–1548). The majority of participants (51.7%, *n* = 201) were classified as low physical activity, 31.9% (*n* = 124) as moderate, and 16.5% (*n* = 64) as high. IPAQ component distributions were also highly non-normal: median vigorous MET-min/week was 0 (IQR 0–0), median moderate MET-min/week was 0 (IQR 0–70), and median walking MET-min/week was 330 (IQR 148.5–693). Median weekday sitting time was 300 min per day (IQR 180–360). A higher proportion of males were classified as high physical activity (43.5% vs. 8.1%), while a higher proportion of females were classified as low activity (58.6% vs. 29.3%). Sample characteristics and fitness profiles are presented in Table 1.

### 3.2. Field-Assessed Functional Capacity Profile

Mean ISWT distance was 439.1 ± 306.0 m (median 360 m, IQR 230–550 m). ISWT distance was positively skewed (Shapiro–Wilk W = 0.840, *p* < 0.001). ISWT distance was higher in males than females (mean 596.7 ± 388.1 m vs. 390.3 ± 257.5 m; Mann–Whitney *U* = 8876, *Z* = −5.08, *p* < 0.001). Rank-based tertile classification produced three near-equal groups: lowest tertile *n* = 128 (32.9%), middle tertile *n* = 133 (34.2%), and highest tertile *n* = 128 (32.9%).

### 3.3. Association Between IPAQ and ISWT

Spearman rank correlations (Table 2) indicated a weak but statistically significant positive association between IPAQ total MET-min/week and ISWT distance (ρ = 0.209, 95% CI 0.112–0.302, *p* < 0.001). All IPAQ intensity domains were positively associated with ISWT distance: vigorous MET-min/week (ρ = 0.151, 95% CI 0.053–0.247, *p* = 0.003), moderate MET-min/week (ρ = 0.143, 95% CI 0.044–0.239, *p* = 0.005), and walking MET-min/week (ρ = 0.176, 95% CI 0.078–0.271, *p* < 0.001). Sitting time was not significantly associated with ISWT distance (ρ = 0.035, 95% CI −0.065 to 0.134, *p* = 0.495).

Sex-stratified analyses were conducted as exploratory subgroup analyses. In females (*n* = 297), total MET-min/week was significantly associated with ISWT distance (ρ = 0.176, 95% CI 0.063–0.284, *p* = 0.002), with additional significant associations for moderate (ρ = 0.175, 95% CI 0.062–0.283, *p* = 0.002) and walking MET-min/week (ρ = 0.125, 95% CI 0.011–0.235, *p* = 0.031). In males (*n* = 92), none of the IPAQ components were significantly associated with ISWT distance (total MET: ρ = −0.035, 95% CI −0.238 to 0.171, *p* = 0.739; vigorous: ρ = −0.092, *p* = 0.380; moderate: ρ = −0.042, *p* = 0.693; walking: ρ = 0.186, *p* = 0.073). Associations were statistically significant among female but not male participants; however, the smaller male subgroup produced less precise estimates, and no formal test of the sex difference in correlation magnitude was conducted, so these sex-stratified findings should be regarded as exploratory. The confidence intervals in the male subgroup all included zero and encompassed both negative and positive associations, indicating that the male estimates were less precise; the absence of statistically significant correlations in males therefore reflects this imprecision and should not be interpreted as evidence of no association.

### 3.4. ISWT Performance Across IPAQ Categories

The Kruskal–Wallis test indicated a statistically significant difference in ISWT distance across IPAQ categories (H (2) = 29.355, *p* < 0.001; Table 3). Bonferroni-adjusted Dunn pairwise comparisons following the Kruskal–Wallis test showed that the overall difference was attributable to the high-activity group: low vs. high (adjusted *p* < 0.001) and moderate vs. high (adjusted *p* = 0.001) differed significantly, whereas low vs. moderate did not (adjusted *p* = 0.162). In summary, IPAQ-SF did not discriminate between low- and moderate-physical-activity categories in terms of ISWT performance; only the high-activity category was statistically distinguishable from the low- and moderate-activity categories.

### 3.5. Classification Agreement and Directional Discordance

Cross-tabulation of IPAQ categories against ISWT fitness tertiles (Table 4) revealed a statistically significant but weak association (χ^2^(4) = 18.764, *p* < 0.001; Cramér’s *V* = 0.155). Cohen’s unweighted kappa indicated slight agreement (κ = 0.083, 95% CI 0.014 to 0.152, *p* = 0.016). The primary ordinal estimate, linear-weighted kappa, was κ_L = 0.137 (95% CI 0.065–0.211), and the quadratic-weighted sensitivity estimate was κ_w = 0.192 (95% CI 0.104–0.279). Allowing partial credit for adjacent-category agreement increased the estimate modestly, but all three kappa specifications fall within the “slight” range of standard benchmarks [28] and concur that classification agreement between IPAQ-SF categories and ISWT-derived fitness tertiles is poor at the individual level. Matching category assignment between IPAQ and the field-assessed fitness tertile occurred for 38.8% of participants (*n* = 151).

Lower-than-tertile IPAQ classification (field-assessed fitness exceeding self-reported activity category) was the most common pattern, occurring in 42.9% of participants (*n* = 167). Higher-than-tertile IPAQ classification was less common, occurring in 18.3% (*n* = 71). The agreement matrix showed that the low IPAQ category exhibited substantial distributional spread across fitness tertiles: 38.8% of low-activity participants fell in the lowest fitness tertile, 35.8% in the middle tertile, and 25.4% in the highest tertile, indicating that a substantial proportion of those classified as low active by self-report achieved field-assessed fitness levels in the middle or highest tertile.

Directional discordance differed significantly by sex (χ^2^(2) = 14.814, *p* < 0.001). Among females, lower-than-tertile IPAQ classification was the dominant pattern (44.1%), followed by matching assignment (41.8%) and higher-than-tertile classification (14.1%). Among males, lower-than-tertile classification occurred in 39.1%, matching assignment in 29.3%, and higher-than-tertile classification in 31.5%.

### 3.6. Exploratory Covariate-Adjusted and Sex-Specific-Tertile Sensitivity Analyses

Exploratory sensitivity analyses showed that the association between IPAQ total MET-min/week and ISWT distance remained statistically significant after adjustment for BMI, age, and sex. The rank-based partial correlation was *r* = 0.144 (*p* = 0.004; *n* = 389), numerically attenuated relative to the unadjusted association (ρ = 0.209) but remaining statistically significant. Exploratory BMI-stratified Spearman correlations were positive in the underweight/normal group (*n* = 197, *r_s* = 0.235, *p* = 0.001) and the obesity group (*n* = 76, *r_s* = 0.248, *p* = 0.028), whereas the correlation in the overweight group was not statistically significant (*n* = 116, *r_s* = 0.160, *p* = 0.084). Because no formal interaction test or comparison of correlation coefficients across BMI categories was performed, these subgroup findings are interpreted descriptively and cautiously.

Because the primary tertiles were derived from the full pooled sample—within which males achieved higher ISWT distances on average—a sensitivity analysis was conducted using ISWT tertiles derived separately within each sex. Under sex-specific tertiles, limited overall classification agreement persisted (unweighted κ = 0.084, *p* = 0.014), and lower-than-tertile IPAQ classification remained the most common pattern overall (43.4%), followed by matching assignment (38.8%) and higher-than-tertile classification (17.7%). The sex difference in directional discordance also persisted and remained highly significant (χ^2^(2) = 29.784, *p* < 0.001): among females, lower-than-tertile 48.5%, matching 39.4%, higher-than-tertile 12.1%; among males, lower-than-tertile 27.2%, matching 37.0%, higher-than-tertile 35.9%. The overall limited agreement therefore persisted when ISWT tertiles were derived separately within sex, and the distribution of directional discordance continued to differ by sex. However, the balance of lower- versus higher-than-tertile IPAQ classification among male participants changed after sex-specific ranking (from predominantly lower-than-tertile under pooled tertiles to predominantly higher-than-tertile under sex-specific tertiles), indicating that sex-specific directional patterns are partly sensitive to the choice of pooled versus sex-specific ISWT cut-points.

## 4. Discussion

This study examined the association, agreement, and directional discordance between self-reported physical activity assessed by IPAQ-SF and field-assessed functional capacity using ISWT among university students in Kuwait. Three principal findings emerge. First, although a statistically significant weak association was observed between IPAQ-derived activity and ISWT distance at the group level, classification agreement at the individual level was poor under unweighted, linear-weighted, and quadratic-weighted kappa specifications, with matching IPAQ and ISWT-tertile category assignment in fewer than four in ten participants. Second, IPAQ-SF did not demonstrate the ability to discriminate between low and moderate physical activity levels in terms of ISWT performance, with only the high-activity category statistically distinguishable from the low- and moderate-activity categories in ISWT performance. Third, directional discordance was sex-patterned: lower-than-tertile IPAQ classification predominated overall, and males showed higher-than-tertile classification at a substantially higher rate than females.

### 4.1. Construct Distinction: Association Versus Agreement

The finding that a statistically significant correlation (ρ = 0.209, *p* < 0.001) coexists with low kappa values (unweighted κ = 0.083; linear-weighted κ_L = 0.137; quadratic-weighted κ_w = 0.192) is not contradictory but reflects a fundamental methodological distinction that is frequently underappreciated in physical activity research. A correlation quantifies directional co-variation between two continuous variables at the group level, while kappa quantifies the degree to which two categorical classification systems assign the same individuals to the same categories. This finding is consistent with Steene-Johannessen et al. [14], who demonstrated that self-report instruments with acceptable group-level correlations can show poor sensitivity and specificity when used to classify individuals as active or inactive. Similar poor individual-level agreement has been observed when comparing self-reported and objectively measured fitness in young adults across different settings [29], and kappa-based analyses in clinical populations confirm that IPAQ-SF category agreement with device-based measures remains negligible regardless of the cut-points applied [30]. Recent work using laboratory cardiopulmonary exercise testing in university students likewise found that IPAQ explained only a small share of variance in measured VO_2_peak [13], consistent with our finding of limited individual-level agreement between IPAQ-SF categories and sample-derived ISWT functional-capacity tertiles. The practical implication is that a significant correlation between IPAQ and ISWT does not validate the use of IPAQ for individual-level fitness classification, and findings from correlation-based validation studies should not be generalised to classification accuracy.

### 4.2. Limited Discriminatory Capacity Between Low and Moderate Activity

A distinctive and practically important finding is the absence of a statistically significant difference in field-assessed functional capacity between participants classified as low and moderate physical activity by IPAQ (Bonferroni-adjusted Dunn pairwise *p* = 0.162), despite a highly significant overall Kruskal–Wallis result. This pattern suggests limited separation between the low and moderate IPAQ categories in relation to ISWT performance, with only the high-activity tier distinguished from the rest; we therefore avoid characterising IPAQ-SF as a strictly binary instrument. This finding aligns with evidence that IPAQ-SF tends to perform most poorly in the moderate-activity range, where the boundaries between categories are most sensitive to reporting inaccuracies and where the MET-based scoring of walking and moderate activity is most susceptible to misinterpretation [11,15].

### 4.3. Direction of Discordance: Lower-than-Tertile Classification Predominates

The predominance of lower-than-tertile IPAQ classification (42.9%) over higher-than-tertile classification (18.3%) warrants careful interpretation, and notably differs from the higher-than-expected-classification pattern most commonly described in Western adult populations [15,16]. IPAQ captures intentional, structured, and perceived activity over the past seven days, whereas ISWT reflects the cumulative physiological consequence of habitual movement, including incidental, occupational, and low-intensity activity that may not be consciously encoded as “exercise” by respondents. This interpretation is supported by evidence that cardiovascular fitness moderates the relationship between self-reported and objective physical activity, with less fit individuals more likely to overreport and fitter individuals more likely to underreport relative to objective measures [17]. Furthermore, recent validation data confirm a pattern of differential bias across the fitness spectrum, where IPAQ-SF underestimates MVPA in low-active individuals and overestimates it in high-active individuals [31]. The finding that 25.4% of those classified as low active achieved the highest field-assessed fitness tertile is consistent with this interpretation and raises the possibility that IPAQ may not fully capture activity that meaningfully contributes to fitness in this population. Several alternative or complementary explanations should also be considered. First, cultural norms may influence how students interpret, label, and report leisure, domestic, incidental, or structured movement [19]; however, the direction of any such reporting bias was not measured in the present study and remains speculative. Second, individual differences in body size and composition, regular non-exercise activity, prior training history, test familiarity, pacing or motivation during the ISWT, and other unmeasured determinants of functional capacity may all contribute to the observed mismatch between self-reported activity and field-test performance. These findings should therefore not be interpreted as evidence of systematic IPAQ overestimation in the conventional sense; rather, they reflect the inherent limitations of comparing a behavioural self-report instrument against a field-based functional capacity measure in this population.

### 4.4. Sex Differences in Directional Discordance

The significant sex difference in directional discordance (*p* < 0.001) adds an important dimension to the interpretation. Males showed higher-than-tertile IPAQ classification at a higher rate than females (31.5% vs. 14.1%), while females showed higher rates of lower-than-tertile classification (44.1% vs. 39.1%). Evidence from multi-country studies demonstrates that sex is a significant moderator of agreement between IPAQ and accelerometry, with males consistently showing larger absolute disagreement than females, particularly for vigorous-intensity activities [32]. The absence of statistically significant IPAQ–ISWT correlations among males should not be interpreted as evidence of no association. The male subgroup was smaller, estimates were less precise (all components *p* > 0.05, all 95% CIs spanning zero), and no formal test of sex differences in correlation magnitude was conducted. These exploratory sex-stratified findings therefore require confirmation in larger, sex-balanced samples. The observed sex-specific pattern may reflect differences in self-report interpretation and the determinants of functional capacity discussed above; these mechanisms were not directly measured and remain speculative. Additionally, contemporary regional evidence indicates that women in Gulf settings face distinct cultural and structural barriers to participation in structured physical activity [19], which may further influence both behaviour and self-reporting patterns.

### 4.5. Covariate-Adjusted Findings

The association between IPAQ total MET-min/week and ISWT distance remained statistically significant after adjustment for BMI, age, and sex, although the estimate was numerically attenuated (rank-based partial correlation *r* = 0.144, *p* = 0.004; *n* = 389). This indicates that the weak IPAQ–fitness association observed at the group level is not fully explained by these demographic and anthropometric covariates, while also confirming that they account for a meaningful share of the unadjusted association. Exploratory BMI-stratified correlations were positive and significant in the underweight/normal and obesity groups but not in the overweight group; because no formal interaction test or comparison of coefficients across BMI categories was performed, these subgroup findings are interpreted descriptively and cautiously and should not be read as evidence that the association differs by BMI category. Together, these exploratory sensitivity analyses support the primary finding of limited individual-level agreement between IPAQ-SF categories and sample-derived ISWT functional-capacity tertiles.

### 4.6. Implications for NCD Surveillance and Risk Profiling

These findings carry direct implications for physical activity surveillance in Kuwait and the broader GCC region, where NCD burden is high and surveillance relies predominantly on self-report questionnaires [20,21]. Because ISWT tertiles were derived from the study sample, these findings concern agreement with relative functional-capacity ranking rather than clinical NCD-risk classification. They show that IPAQ-SF and a field-based functional-capacity marker do not consistently classify the same individuals into matched strata. When IPAQ categories were compared with ISWT tertiles, 61.2% of participants had non-matching category assignments; when this occurs in a population compared against field-assessed fitness rankings, IPAQ-SF and field-based functional-capacity measures should be regarded as related but non-interchangeable for individual-level categorisation. The findings support a complementary use of health markers: self-reported physical activity remains valuable for capturing behavioural patterns and exposure context, while an accessible field-based functional-capacity marker such as the ISWT provides additional performance-based information that does not require laboratory equipment [23,24]. Combining behavioural and performance-based information may support intervention targeting and health monitoring in university and community settings. Replication in multi-site, nationally representative samples across GCC countries is needed before these findings can support population-level surveillance recommendations.

### 4.7. Strengths and Limitations

A key strength of this study is its focus on classification agreement and directional discordance rather than correlation alone, providing an assessment of the IPAQ–fitness relationship that is more directly relevant to clinical and surveillance decision-making. The use of the ISWT as a standardised, field-based fitness marker makes the findings applicable to resource-limited settings, and the sex-stratified analysis adds interpretive depth.

Several limitations must be acknowledged. The cross-sectional design precludes causal inference. Although weighted kappa is conventionally preferred for ordinal categories, the unweighted, linear-weighted (primary ordinal estimate), and quadratic-weighted kappa coefficients all fell within the “slight” range, consistently supporting the conclusion that classification agreement at the individual level is poor. Although the ISWT is a validated and widely used field-based measure of functional exercise capacity [25], it is not a direct measure of maximal cardiorespiratory fitness (e.g., VO_2_max), and ISWT tertiles should be interpreted as a sample-derived proxy for relative fitness ranking rather than an absolute or clinically defined gold standard. Several variables that may influence ISWT performance independently of recent self-reported activity—including body composition beyond BMI, smoking status, sports participation, prior training history, test familiarity, motivation, and overall health status—were not incorporated into the present analysis; some were available in the broader parent study while others were not measured, and these together with genetic and motivational factors may contribute to the observed IPAQ–ISWT discordance. BMI, age, and sex were incorporated in exploratory covariate-adjusted sensitivity analyses (Section 3.6), and the IPAQ–ISWT association persisted after adjustment; a fuller covariate model lies beyond the scope of the present agreement-focused analysis and is a priority for subsequent analyses within this series. The absence of device-based accelerometry means that this study quantifies IPAQ–fitness discordance rather than IPAQ–activity discordance, which limits direct comparison with traditional IPAQ validation studies. Additional limitations include the convenience sampling approach (with associated self-selection bias), the marked female-to-male imbalance (which yields less precise sex-specific estimates), the predominantly Kuwaiti national composition of the sample, and the recognised susceptibility of IPAQ-SF to social desirability bias. Cultural norms may influence how students interpret and report structured, incidental, or domestic movement; however, the direction of any such reporting bias was not measured in this study and remains speculative. The convenience sample from a single university may limit generalisability to other GCC or Middle East and North Africa (MENA) populations, and replication in multi-site, nationally representative, sex-balanced samples is required.

## 5. Conclusions

Self-reported physical activity assessed by IPAQ-SF shows a weak but statistically significant association with field-assessed functional capacity at the group level among university students in Kuwait; however, classification agreement at the individual level is poor across unweighted, linear-weighted, and quadratic-weighted kappa specifications (κ = 0.083, κ_L = 0.137, and κ_w = 0.192, respectively). IPAQ-SF did not demonstrate the ability to discriminate between low and moderate physical activity levels in terms of ISWT performance, suggesting limited separation between the low and moderate IPAQ categories in relation to ISWT performance. Lower-than-tertile classification predominated overall, and directional discordance differed by sex; however, these sex-specific findings remain exploratory because the convenience sample was predominantly female and the male subgroup was comparatively small. In GCC settings where NCD burden is high and physical activity surveillance relies heavily on self-report, incorporating accessible field-based functional-capacity assessment alongside questionnaire tools may add complementary performance-based information. These findings concern agreement with sample-derived relative functional-capacity tertiles, rather than validated clinical categories of cardiorespiratory fitness or NCD risk. Replication in multi-site, nationally representative samples is required before population-level surveillance recommendations can be generalised.

## Figures and Tables

**Table 1:** Physical activity and field-assessed functional capacity profile of the study sample (*N* = 389).

Variable	Overall (*N* = 389)	Female (*n* = 297)	Male (*n* = 92)
Self-reported physical activity (IPAQ-SF)			
Total MET-min/week, median (IQR)	540 (198–1548)	450 (148.5–1069.5)	1576 (588.4–3888.8)
IPAQ category: Low, *n* (%)	201 (51.7)	174 (58.6)	27 (29.3)
IPAQ category: Moderate, *n* (%)	124 (31.9)	99 (33.3)	25 (27.2)
IPAQ category: High, *n* (%)	64 (16.5)	24 (8.1)	40 (43.5)
Vigorous MET-min/week, median (IQR)	0 (0–0)	0 (0–0)	360 (0–2400)
Moderate MET-min/week, median (IQR)	0 (0–70)	0 (0–0)	0 (0–360)
Walking MET-min/week, median (IQR)	330 (148.5–693)	297 (108.9–693)	462 (247.5–693)
Sitting time, min/day, median (IQR)	300 (180–360)	300 (180–360)	300 (195–405)
Field-assessed functional capacity (ISWT)			
ISWT distance (m), mean ± SD	439.1 ± 306.0	390.3 ± 257.5	596.7 ± 388.1
ISWT distance (m), median (IQR)	360 (230–550)	310 (210–500)	445 (322.5–802.5)
ISWT tertile 1 (lowest), *n* (%)	128 (32.9)	112 (37.7)	16 (17.4)
ISWT tertile 2 (middle), *n* (%)	133 (34.2)	101 (34.0)	32 (34.8)
ISWT tertile 3 (highest), *n* (%)	128 (32.9)	84 (28.3)	44 (47.8)

IQR, interquartile range; IPAQ-SF, International Physical Activity Questionnaire—Short Form; ISWT, Incremental Shuttle Walk Test; MET, metabolic equivalent task; SD, standard deviation. ISWT tertiles were derived from the full pooled sample; sex-specific cells report the number (and percentage within each sex) of participants falling into each pooled tertile. Because males achieved higher ISWT distances on average, a higher proportion of males fall in the highest pooled tertile.

**Table 2:** Spearman rank correlations between IPAQ components and ISWT distance (*N* = 389), overall and sex-stratified.

IPAQ Variable	Overall ρ (95% CI)	Overall *p*	Female ρ (95% CI; *p*)	Male ρ (95% CI; *p*)
**Total MET-min/week**	**0.209 (0.112, 0.302)**	**<0.001**	**0.176 (0.063, 0.284); 0.002**	−0.035 (−0.238, 0.171); 0.739
**Vigorous MET-min/week**	**0.151 (0.053, 0.247)**	**0.003**	0.104 (−0.010, 0.215); 0.073	−0.092 (−0.291, 0.115); 0.380
**Moderate MET-min/week**	**0.143 (0.044, 0.239)**	**0.005**	**0.175 (0.062, 0.283); 0.002**	−0.042 (−0.244, 0.165); 0.693
**Walking MET-min/week**	**0.176 (0.078, 0.271)**	**<0.001**	**0.125 (0.011, 0.235); 0.031**	0.186 (−0.020, 0.376); 0.073
Sitting time (min/day)	0.035 (−0.065, 0.134)	0.495	0.035 (−0.079, 0.149); 0.543	−0.048 (−0.250, 0.158); 0.649

IPAQ, International Physical Activity Questionnaire; ISWT, Incremental Shuttle Walk Test; MET, metabolic equivalent task; 95% CI computed via Fisher’s z-transformation. Statistically significant correlations (*p* < 0.05) are in bold.

**Table 3:** ISWT performance across IPAQ physical activity categories (*N* = 389).

IPAQ Category	*n*	Mean ± SD (m)	Median (IQR) (m)	Mean Rank (KW)
Low	201	371.2 ± 242.4	310 (210–460)	172.76
Moderate	124	438.9 ± 288.5	365 (222.5–570)	197.48
High	64	652.7 ± 408.1	495 (362.5–927.5)	260.04
Overall	389	439.1 ± 306.0	360 (230–550)	H (2) = 29.355, *p* < 0.001

IPAQ-SF categories were assigned using the standard IPAQ scoring algorithm incorporating activity type, reported days, duration, and total MET-min/week; categories were not defined solely by total MET-min/week thresholds. Bonferroni-adjusted Dunn pairwise comparisons: Low vs. High adjusted *p* < 0.001; Moderate vs. High adjusted *p* = 0.001; Low vs. Moderate adjusted *p* = 0.162 (NS). IQR, interquartile range; IPAQ, International Physical Activity Questionnaire; ISWT, Incremental Shuttle Walk Test; KW, Kruskal–Wallis; MET, metabolic equivalent task; NS, not significant; SD, standard deviation.

**Table 4:** Agreement and directional discordance between IPAQ categories and ISWT functional-capacity tertiles (*N* = 389).

IPAQ Category	ISWT Tertile 1 (Lowest) *n* (%)	ISWT Tertile 2 (Middle) *n* (%)	ISWT Tertile 3 (Highest) *n* (%)	Total *n* (%)
Low	78 (38.8)	72 (35.8)	51 (25.4)	201 (51.7)
Moderate	40 (32.3)	40 (32.3)	44 (35.5)	124 (31.9)
High	10 (15.6)	21 (32.8)	33 (51.6)	64 (16.5)
Total	128 (32.9)	133 (34.2)	128 (32.9)	389 (100)

χ^2^(4) = 18.764, *p* < 0.001; Cramér’s V = 0.155. Percentages within IPAQ categories (rows) are shown. Cohen’s unweighted κ = 0.083 (95% CI 0.014 to 0.152, *p* = 0.016); linear-weighted κ_L = 0.137 (95% CI 0.065–0.211, primary ordinal estimate); quadratic-weighted κ_w = 0.192 (95% CI 0.104–0.279). Bootstrap percentile 95% CIs (10,000 resamples, seed 20260622). Directional discordance summary: matching category assignment = 38.8% (*n* = 151); lower-than-tertile IPAQ classification = 42.9% (*n* = 167); higher-than-tertile IPAQ classification = 18.3% (*n* = 71). Sex-stratified: females—lower-than-tertile 44.1%, matching 41.8%, higher-than-tertile 14.1%; males—lower-than-tertile 39.1%, matching 29.3%, higher-than-tertile 31.5% (χ^2^(2) = 14.814, *p* < 0.001). IPAQ, International Physical Activity Questionnaire; ISWT, Incremental Shuttle Walk Test.

## Data Availability

The datasets generated and analysed during the current study are not publicly available due to ethical and legal restrictions imposed by the Kuwait University Health Sciences Centre Ethics Committee (Approval No. VDR/EC-2025-114), which requires institutional oversight for data sharing. Data are available from the corresponding author on reasonable request, subject to approval by the Ethics Committee.

## Abbreviations

The following abbreviations are used in this manuscript:

CRF	Cardiorespiratory fitness
CI	Confidence interval
GCC	Gulf Cooperation Council
IPAQ	International Physical Activity Questionnaire
IPAQ-SF	International Physical Activity Questionnaire—Short Form
IQR	Interquartile range
ISWT	Incremental Shuttle Walk Test
MET	Metabolic equivalent task
MVPA	Moderate-to-vigorous physical activity
NCD	Non-communicable disease
PAR-Q+	Physical Activity Readiness Questionnaire (revised)
SD	Standard deviation
VO_2_max	Maximal oxygen uptake
